# Prenatal cocaine exposure alters alpha2 receptor expression in adolescent rats

**DOI:** 10.1186/1471-2202-7-33

**Published:** 2006-04-18

**Authors:** Rosemarie M Booze, David R Wallace, Janelle M Silvers, Barbara J Strupp, Diane M Snow, Charles F Mactutus

**Affiliations:** 1 Department of Psychology, University of South Carolina Columbia, SC 29208, USA; 2 Department of Pharmacology, Physiology and Neuroscience, University of South Carolina Columbia, SC 29208, USA; 3 Department of Pharmacology and Physiology, Oklahoma State University, College of Osteopathic Medicine Tulsa, OK 74017-1898, USA; 4 Division of Nutritional Sciences and Department of Psychology, Cornell University Ithaca, NY 14853-6301, USA; 5 Department of Anatomy and Neurobiology, The University of Kentucky, Lexington, KY 40536-0298, USA

## Abstract

**Background:**

Prenatal cocaine exposure produces attentional deficits which to persist through early childhood. Given the role of norepinephrine (NE) in attentional processes, we examined the forebrain NE systems from prenatal cocaine exposed rats. Cocaine was administered during pregnancy via the clinically relevant intravenous route of administration. Specifically, we measured α_2_-adrenergic receptor (α_2_-AR) density in adolescent (35-days-old) rats, using [^3^H]RX821002 (5 nM).

**Results:**

Sex-specific alterations of α_2_-AR were found in the hippocampus and amygdala of the cocaine-exposed animals, as well as an upregulation of α_2_-AR in parietal cortex.

**Conclusion:**

These data suggest that prenatal cocaine exposure results in a persistent alteration in forebrain NE systems as indicated by alterations in receptor density. These neurochemical changes may underlie behavioral abnormalities observed in offspring attentional processes following prenatal exposure to cocaine.

## Background

Recently, noradrenergic systems have been identified as a potential teratogenic target underlying the functional effects of prenatal cocaine [1-3]. However, information regarding the consequences of prenatal cocaine on the development of noradrenergic receptors is relatively sparse. NE is present early in brain development and regulates important aspects of prenatal brain development, including neural migration and synaptogenesis [4,5]. Thus, the ability of cocaine to inhibit NE reuptake has potentially profound effects on the developing nervous system and function of NE systems.

Previous investigations into the effects of prenatal cocaine exposure on catecholaminergic receptors have, for the most part, focused on the long-term effects of exposure on dopaminergic [6-10] and serotonergic [11-14] receptor systems. Fewer studies have examined the noradrenergic receptor family following prenatal cocaine exposure. The neurophysiological effects of NE are mediated by three types of receptors: α_1_, α_2 _and β. The α_2 _adrenergic receptors are present very early in development, in some brain areas as early as E15 [4]. Prenatal exposure to cocaine has been found to elevate the density of α_2 _adrenergic receptors in the cerebellum and forebrain [15]. Henderson et al [16] reported that cortical α_2 _adrenergic receptor density was unchanged in male rat pups following prenatal cocaine exposure. However, these studies did not differentiate between male and female offspring and used homogenate binding techniques. Moreover, cocaine was administered via the subcutaneous route into the dams, and therefore these effects likely occurred in the presence of potential nutritional and stress confounds [17].

Previous studies from this laboratory and others have demonstrated that the IV route of cocaine administration to pregnant rats produces functional alterations in attentional processes [2,18-21]. Subtle, context-specific sex differences in attentional tasks following prenatal cocaine have been reported in a number of these studies [18-21]. The neurological basis of such attentional deficits is complex and likely mediated by several neurotransmitter systems. Several studies have assessed the involvement of norepinephrine specifically in attentional processes. The development of the heart rate orienting response in preweaning rats, a task used to measure attention to a novel stimus, is dependent upon norepinephrine, but not dopamine or serotonin [22,23]. Alterations in the heart rate orienting response of cocaine-treated offspring suggest early impairments in noradrenergic systems [2,4,18]. Direct evidence of the effects of cocaine on norepinephrine systems has been provided by Snow et al. [24], in which cocaine was found to directly inhibit process outgrowth in locus coeruleus (LC) neurons.

Altered attention has been reported in 6 year old children gestationally exposed to moderate levels of cocaine [25]. The impairment in automated vigilance task in 6 year olds most likely reflects a deficit in sustained attention and one that also contained an accuracy component (commission vs. omission errors). The NE system is thought to be critically involved in the regulation of attention [26-31]. That is, the activation of NE serves to filter out distracting or competing stimuli and plays a role in selective attention in rats [32]. A recent study reports that rats exposed to prenatal cocaine are more sensitive to impairment of selective attention by idazoxan, an α2 adrenergic receptor agonist [1]. These findings, and reported deficits in vigilance/orienting performance of young rats prenatally exposed to IV cocaine [2,18] suggest that NE plays an important role in attention and in long-term cocaine impairments [19-21]. The mechanisms of cocaine-induced disruption of NE developmental patterns and the relationship between these patterns and the attentional alterations remain to be determined.

For the most part, the effects of prenatal cocaine exposure have been assessed either immediately, during the preweaning period, or long-term, i.e. into mature adulthood. Recently, the adolescent period has been recognized as a period of vulnerability to the effects of drugs of abuse [33]. Exposure to drugs during early development may alter critical neural development, producing long-term effects on sexual maturation and sex-specific behaviors which are manifested during the adolescent period [34,35]. Thus, the adolescent period may represent a unique developmental time frame in which to examine the neurological effects of prenatal drug exposure.

In the present studies we examined potential sex-dependent alterations in α_2 _adrenergic receptor density and function in adolescent rats following prenatal cocaine exposure. The idazoxan derivative, RX821002, was used to detect α_2 _adrenergic receptors. RX821002 is a highly selective antagonist that identifies all the α_2 _adrenergic receptor subtypes with similar affinity [36]. We first used this well-characterized ligand to determine α_2 _receptor density and binding affinity in adolescent rats that received cocaine *in utero*. We then commenced a more detailed receptor autoradiographic study of the hippocampus, parietal cortex, amygdala, pyriform cortex and hypothalamus to determine whether sex differences in α_2 _receptors were present in adolescent rats prenatally exposed to cocaine. These studies were designed as part of a larger effort to understand the neurobiological basis of the previously reported attentional dysfunction in rats prenatally exposed to cocaine [1,2,19-21,37].

## Results

### Weight/growth parameters

The mean offspring body weights (on P35) were unaffected by prenatal cocaine exposure. Previous reports have shown that this regimen of in utero cocaine treatment had no effects on maternal weight gain, litter size, gestational length, sex ratio, offspring weight on postnatal day 1, or pup survival [1,2,38].

### α2-adrenergic receptor density: tissue homogenates

**Figure 1:**
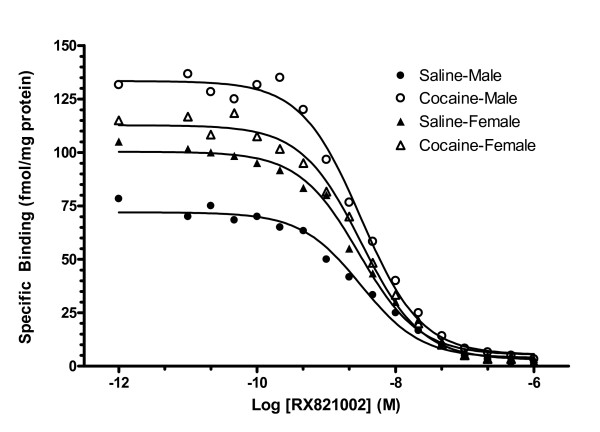
Representative competition ("cold saturation") analysis curve measured in hippocampus from male and female offspring exposed to cocaine in utero. Analysis was performed by the addition of 2 nM [^3^H] RX821002 added to tubes containing unlabeled RX821002 (1 pM – 1μM) or 10μM phentolamine (nonspecific binding). Data were best fit to a single-site model with hill slopes (n_H_) approaching unity. Calculated K_d _and B_MAX _values are expressed in Table 1.

Results of tissue homogenate binding studies are displayed in Table 1 and Figure 1. The affinity of RX821002 for α_2_-adrenergic receptors did not differ significantly by sex or prenatal cocaine exposure In all groups, the displacement of [^3^H]RX821002 by unlabeled RX821002 was close to unity (0.94 ± 0.05 – 1.09 ± 0.16) suggesting displacement from a single binding site. Labeling of α_2_-adrenergic receptors by [^3^H]RX821002 was sex-dependent within the saline group with females displaying 36.2% higher density of α_2_-adrenergic receptors than males. The number of α_2_-adrenergic receptors labeled with 2.0 nM [^3^H]RX821002 following prenatal cocaine exposure was significantly increased [F(1, 20) = 5.0; *P *≤ 0.04) in both males and females. This effect was most profound in cocaine-exposed males, as they exhibited a 58.1% increase in α_2_-adrenergic receptor density, relative to saline treated males [F(1, 20) = 6.9; *P *≤ 0.02). Thus, prenatal cocaine produced alterations in α_2_-adrenergic receptor density in the hippocampus without changes in binding affinity.

### α2-adrenergic receptor density: autoradiography

**Figure 2:**
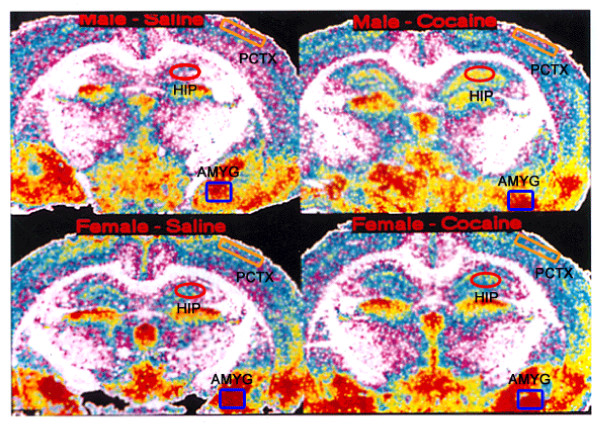
Representative false-color (RGB) autoradiograms of [^3^H]RX821002 binding density in brain slices from adolescent males and females exposed to either saline or cocaine *in utero*. Areas of low binding density appear white to light purple, areas of moderate binding appear blue to yellow, and areas of high binding density appear dark red. Measurements were taken from hippocampus (stratum lacunosum-moleculare of CA1 near the hippocampal fissure, as approximately marked by a red oval), parietal cortex (layer II, as approximately marked by an orange rectangle), and amygdala (central nucleus, as approximately marked by a blue square).

Representative [^3^H]RX821002 autoradiograms are depicted in Figure 2. Tissue sections incubated with 2 nM [^3^H]RX821002 exhibited both sex- and cocaine-induced alterations in α_2_-adrenergic receptor density. Following prenatal exposure to cocaine, the density of α_2_-adrenergic receptors demonstrated a significant interaction between region, sex and cocaine [F(4, 208) = 4.7; *P *≤ 0.007]. The binding of [^3^H]RX821002 to α_2_-adrenergic receptors exhibited significant differences between region and sex within the saline group [F(4, 208) = 10.2; *P *≤ 0.0001]. Prenatal cocaine exposure significantly affected the density of α_2_-adrenergic receptors in male rats across the regions examined [F(4, 208) = 4.0; *P *≤ 0.01].

**Figure 3:**
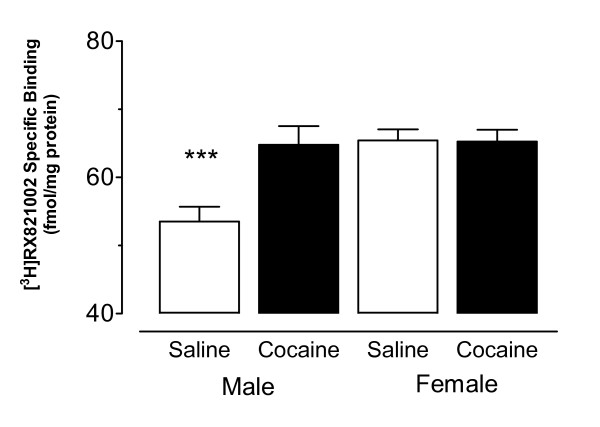
Density of α_2_-adrenergic receptors in laconosum-moleculare layer of hippocampal area CA1 as measured by autoradiography. Data are expressed as mean density (± S.E.M.). In control animals prenatally exposed to saline, the density of hippocampal CA1 α_2_-adrenergic receptors in male rats was significantly [F(1, 52) = 23.2; P < 0.0001] lower than the density of α_2_-adrenergic receptors observed in female controls. Prenatal cocaine exposure abolished this sex difference in hippocampal α_2_-adrenergic receptors by increasing binding 21% in male offspring, whereas α_2_-adrenergic receptors did not significantly differ by drug exposure in females. n = 14 per group.

In control animals prenatally exposed to saline, the density of hippocampal CA1 α_2_-adrenergic receptors in female rats was significantly [F(1, 52) = 23.2; *P *≤ 0.001] higher than the density of α_2_-adrenergic receptors observed in male controls (Figure 3). In the area CA1 of the hippocampus, a significant interaction between drug treatment and sex was observed [F(1, 52) = 20.00; *P *≤ 0.001]. Similar to tissue homogenates, laconosum-moleculare layer of area CA1 hippocampal α_2_-adrenergic receptors were upregulated 21% in male offspring following cocaine exposure [F(1,52) = 6.8; P < 0.01], whereas α_2_-adrenergic receptors did not significantly differ in females.

**Figure 4:**
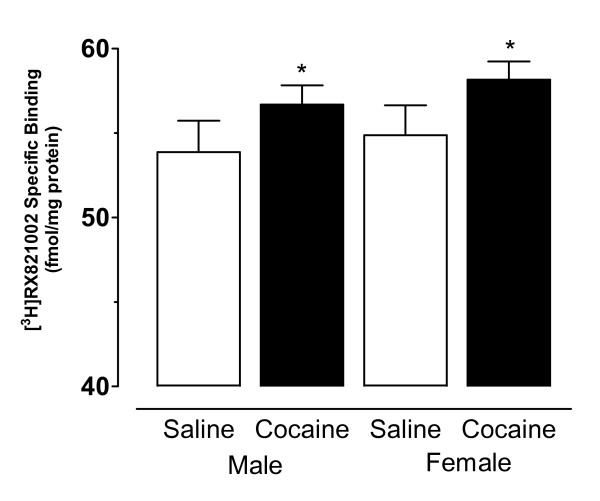
Density of α2 adrenergic receptors in parietal cortex (layer II) as measured by autoradiography. Data are expressed as mean density (± S.E.M.). In the parietal cortex, prenatal cocaine exposure increased α2 adrenergic receptors in both sexes [F(1, 52) = 4.1; P < 0.05]. n = 14 per group.

**Figure 5:**
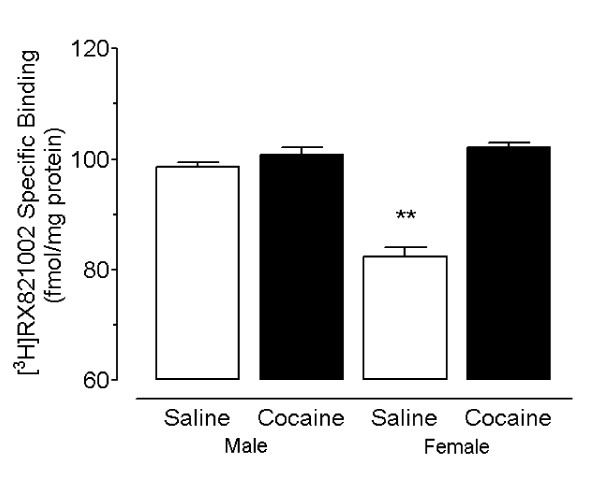
Density of α_2_-adrenergic receptors in amygdala (central nucleus) as measured by autoradiography. In control animals, the density of α_2_-adrenergic receptors was significantly lower [F(1, 52) = 10.9; P < 0.01] in females than the density of α_2_-adrenergic receptors observed in male controls. Prenatal cocaine exposure abolished this sex difference in amygdala α_2_-adrenergic receptors by increasing binding by 19% in female offspring, whereas α_2_-adrenergic receptors did not significantly differ because of cocaine exposure in males. n = 14 per group.

Of the other brain regions examined, only the parietal cortex and central nucleus of the amygdala exhibited alterations in α_2_-adrenergic receptor density following prenatal cocaine exposure. In the parietal cortex, a significant effect of drug was observed [F(1, 52) = 4.1; *P *≤ 0.05], with no effect of sex or interaction between sex and prenatal drug treatment (Figure 4). In the amygdala, the density of α_2_-adrenergic receptors was significantly [F(1, 52) = 10.9; *P *≤ 0.002] higher in males compared to females from the saline group (Figure 5). Prenatal cocaine exposure increased the number of α_2_-adrenergic receptors in the amygdala from female rats compared to saline treated females [F(1, 52) = 7.2; *P *≤ 0.01] such that the density of α_2_-adrenergic receptors in this region was similar to the density observed in male rats (Figure 5). There were no effects of either sex or prenatal drug exposure on the density of α_2_-adrenergic receptors in either the pyriform cortex or periventricular nucleus of the hypothalamus.

## Discussion

The present study found alterations in α_2 _adrenergic receptor density in the adolescent brain subsequent to prenatal cocaine exposure. Adolescence represents a period of vulnerability for substance abuse and age-dependent sensitivity to drugs. Receptor alterations expressed during adolescence may modify essential transitions necessary for producing normal adult brain function [39]. Prenatal IV cocaine exposure appears to alter normal development of the NE receptor systems, leading to altered adolescent NE brain systems.

In general, catecholamine receptor density and function appear to be particularly plastic during the adolescent period. For example, dopamine D1 and D2 receptors are overproduced and eliminated (over 40%) in male rats during adolescence [40]. The distribution of α2 adrenergic receptors has been reported to generally resemble adult patterns by P28 [41]; however, functional studies have demonstrated decreased depolarized release of NE and a greater capacity for NE reuptake in the hypothalamus (with a potential shift in hypothalamic alpha receptor subtype) is present in rats during the adolescent period [42]. Sex differences in NE content have also been reported in several brain regions in adolescent animals (P33) [43]. Prenatal cocaine exposure has been shown to result in increased levels of NE in the preoptic region of male, but not female, adult rats [44]. Previous reports of α2 adrenergic receptor distribution used pooled male and female rat brains without presenting statistical analysis on sex differences [41]. Furthermore, the number of animals used from each litter was unclear. Therefore the current studies are the first to report baseline sex differences in α2 adrenergic receptor density in adolescent animals, as well as alterations in density related to prenatal cocaine exposure in this age group.

Prenatal cocaine exposure may alter α2 adrenergic receptors density by affecting development of the LC. The entire NE input to the rat hippocampus is provided by LC neurons, primarily from the dorsal one-third of the LC [45,46]. In early LC development, approximately 7% of the neurons are generated on GD11, 75% on GD12 and 18% on GD13 [47]. LC neurons and their processes synthesize NE as early as 12–14 days gestation [48] well before synaptogenesis is underway in the terminal fields. Efferent LC fibers first appear in the neocortical terminal fields on GD16 and these fibers are proposed to play a major role in induction and differentiation of neural tissue [49]. Hippocampal neurons are generated three days after the LC neurons [48]. The late gestational period of the rat is distinguished by continued fiber organization and ramification in the LC terminal fields [50]. A recent study, using the same cocaine dose and route of administration used in the current study, demonstrated that prenatal cocaine exposure during the development of LC neurons inhibits the growth of LC neuritis [24]. Cocaine binding sites are evident in the fetal brain as early as GD15 and by GD20 the Kd of [^3^H]cocaine binding is similar to the Kd values observed in adulthood [51]. Thus, our prenatal cocaine exposure covers the period of LC neuronal genesis and NE axonal proliferation in the terminal fields, during which time these systems are sensitive to disruption by cocaine.

Adolescent brain development may represent a critical developmental stage in which prenatal cocaine effects may be expressed in a sex-dependent manner. Our findings of α_2 _adrenergic receptor upregulation in male rat hippocampus may be a compensatory response to cocaine-mediated increases in norepinephrine concentration during prenatal development. Interestingly, female offspring did not show a similar robust increase in α_2 _adrenergic receptor density in the hippocampus. However, in the amygdala the α_2 _adrenergic receptors were increased in females subsequent to prenatal cocaine exposure. In other regions such as the pyriform cortex, no changes attributable to cocaine were found. Our findings suggest sex-specific expression of cocaine-mediated alterations displayed in adolescence. Sex-specific alterations in receptor density may be restricted to particular brain regions, differ in directionality, and may not reflect global brain alterations.

## Conclusion

In summary, IV exposure of pregnant rats to cocaine produced persistent, sex-specific alterations in the NE systems of adolescent offspring. Disruption of forebrain NE systems during the prenatal period might be the neurobiological basis for a number of functional disturbances occurring as a consequence of prenatal cocaine exposure.

## Methods

### Animals

Nulliparous female Sprague-Dawley rats were obtained from Harlan Sprague-Dawley, Inc. (Indianapolis, IN) at approximately 10–12 weeks of age (225–249 g), placed into quarantine for one week and subsequently moved to the animal colony. The animals were maintained according to NIH guidelines in AAALAC accredited facilities. Food (Pro-Lab Rat, Mouse, Hamster Chow No. 3000) and water were available ad libitum. The animal colony was maintained at 21 ± 2°C and 50 ± 10% relative humidity and a 12 hr light: 12 hr dark cycle with lights on at 07:00 h (EST).

### Surgery

Half of the animals were surgically implanted with vascular catheters (as described below) and the remaining animals served as surrogate dams for the prenatal vehicle and cocaine treated pups. Catheterization was performed as previously described [38]. In brief, the animals were anesthetized with a mixture of ketamine hydrochloride (100 mg/kg/ml) and xylazine (3.3 mg/kg/ml) and a sterile Intracath IV catheter with a Luer-lock injection cap (Medex) was implanted dorsally in a subcutaneous pouch. The distal end of the catheter was inserted into the left jugular vein and advanced towards the heart. Animals were kept under periodic postoperative observation and returned to the vivarium upon recovery from anesthesia. Beginning on the day following surgery, the catheters were flushed daily with approximately 0.2 ml of 2.5% heparinized saline. The animals were observed for any signs of discomfort or behavioral distress. Complete anesthesia, surgical, recovery and postoperative records were maintained for each animal.

### Animal mating

At 10–12 days following surgery, the females were group housed with males for breeding. Females were checked daily for vaginal cytology and the presence of sperm. Sperm positive females were considered at gestation day 0 (GD0) and individually housed in plastic cages with Sani-chip™ bedding throughout pregnancy and lactation.

### Drug treatment

The catheterized, pregnant animals were randomly assigned to one of two groups that received either saline or 3.0 mg/kg cocaine. This dose of cocaine, delivered IV, was chosen based on reports that 1) it produces a peak arterial level in the male rat not significantly different from peak levels following administration of 32 mg of cocaine IV to humans [52,53], 2) the acute heart rate and blood pressure responses in late gestation pregnant rats are similar to those produced in a variety of other species (dog, monkey, humans; [54]), and 3) under experimental conditions, this dose is self-administered by users multiple times in a 2.5 hr session [55] Cocaine was administered as an IV bolus injection delivered in a volume of 1.0 ml/kg (15 sec) followed by flushing (15 sec) with 0.2 ml heparinized (2.5%) saline (i.e., the approximate volume of the catheter). Surrogate dams received neither drug treatment nor the daily handling associated with drug treatment. Cocaine and saline injections were administered 1x day from GD8-GD14 and 2x day from GD15-GD21.

### Offspring treatment

All pregnant rats were checked twice daily for pups. Day of birth was defined as postnatal day 0 (P0). On P1, litters were weighed and culled to 8 pups with an equal number of males and females. Each pup was tattooed for identification and the culled litters of all catheterized dams were fostered to surrogate dams that had delivered within 24 h. Thus, no pups were raised by their biological mother or exposed to drug during the postnatal period. Pups were reared in their surrogate mothers' cage until weaning at P21, at which time offspring were group housed in same sex pairings. One male and one female from each litter were sacrificed on P35 for homogenate binding and autoradiographic analysis. Thus, each experiment included pups from 14 different litters.

### α2-adrenergic receptor homogenate binding

Hippocampal tissue was collected for α_2_-adrenergic receptor binding from one male and one female from each litter on P35. Animals were killed by rapid decapitation and the brains removed. The hippocampi were dissected and immediately frozen in liquid nitrogen. Frozen hippocampal tissue was weighed and homogenized in 20 volumes/weight ice-cold 25 mM glycylglycine (GLYGLY) buffer (pH = 7.6). Crude homogenates were centrifuged at 20,000X g for 15 min at 4°C. The pellet was resuspended in 200 volumes 25 mM GLYGLY buffer (pH = 7.6) and the resulting crude membrane preparation was used for binding assays at a final protein content of approximately 0.6–0.7 mg/ml. Protein contents were determined by the Bradford [56] method (BioRad, Richmond, CA).

Cold saturation homogenate binding assays were performed as previously described [36]. Briefly, for the binding of [^3^H]RX821002 (58 Ci/mmol; Amersham, Arlington Heights, IL), 50 μl of labeled drug (2 nM) and 50 μl of assay buffer containing one of seventeen concentrations (10^-12 ^to 10^-6^) of unlabeled RX821002 were added together and allowed to equilibrate to room temperature (22°C). Binding was initiated by addition of 900 μl of tissue (0.6–0.7 mg of protein) and allowed to incubate to equilibrium at 22°C for 60 min. Nonspecific binding was defined as [^3^H]RX821002 bound in the presence of 10 μM phentolamine. Binding was terminated by filtration under reduced pressure with a Brandel Tissue Harvestor (Gaithersburg, MD) followed by a 15 sec wash with ice-cold GLYGLY buffer onto GF/B glass fiber filters that had been presoaked for 2 hours with buffer containing 0.3% polyethyleneimine. Filters were dried overnight and analysis of radioligand bound was accomplished by scintillation spectrophotometry (40–50% efficiency).

### α2-adrenergic receptor autoradiography

For preparation of tissue sections, animals were killed by rapid decapitation and the brains were carefully removed from the cranium, blocked and immediately frozen on powdered dry ice. The frozen tissue blocks were cryostat-sectioned (-20°C, 20 μm thick) in the standard coronal plane. Sections were collected at -3.3–3.8 mm relative to Bregma (plates 31–33 in Paxinos and Watson, [57]). Two additional adjacent sections were collected either for Nissl staining or acetylcholinesterase (AchE) staining [58] to aid in identification of the subregional structures. All sections were stored desiccated at -80°C prior to processing.

Frozen tissue sections were thawed and brought to room temperature (22°C; 5 min) followed by preincubation in 25 mM GLYGLY buffer (pH = 7.6) for 5 min. Slides were transferred into incubation vials containing 2 nM [^3^H]RX821002 and incubated at 22°C for 90 min. Nonspecific binding was defined by 10 μM phentolamine. Binding was terminated by transfer of slides into 4°C buffer and washing 2 × 2 min. Slides were then quickly rinsed in 4°C distilled water to remove buffer salts, dried under a stream of cool air and stored desiccated overnight under vacuum. Dried, labeled, tissue sections and [^3^H]microscales (Amersham, Arlington, IL) were exposed to tritium sensitive film (Hyperfilm, Amersham) for 14 days at room temperature in light-tight X-ray film cassettes. Films were developed using Kodak D-19 developer and Kodak rapid fixer.

Autoradiographic images were examined using the MCID-4 computerized image analysis system (Imaging Research, Ontario, Canada). The use of adjacent Nissl and AChE stained slides allowed for the determination of [^3^H]RX821002 binding in specific subregions of the hippocampus (stratum lacunosum-moleculare of CA1 near the hippocampal fissure). Binding density was also determined for the parietal cortex (layer II), amygdala (central nucleus), pyriform cortex (layer II-III) and hypothalamus (periventricular nucleus). These brain regions have high levels of α_2 _adrenergic receptors [59]. Adjacent sections, incubated with 10 μM phentolamine (nonspecific binding; < 10% total binding), were aligned with total binding sections and digitally subtracted to obtain "specific" binding images. Regional optical density data are expressed as fmol/mg weight [60,61].

### Data analysis

Data were examined by ANOVA ([62]; BMDP statistical software, release 7, Los Angeles, CA, 1993). The Greenhouse-Geiser df correction factor was used for violations of compound symmetry [63]. An α level of *p *< 0.05 was the significance level set for rejection of the null hypothesis. Analysis of K_D _and B_MAX _values were determined using the GraphPAD-PRISM nonlinear curve fitting program (GraphPAD, San Diego, CA).

### Chemicals

Cocaine HCl was purchased from Sigma Chemical Co. (St. Louis, MO) and was dissolved in sterile isotonic saline at the indicated concentrations based on the weight of the salt. All cocaine solutions were prepared immediately prior to use in a volume of 1 ml/kg. All other buffer chemicals were obtained from Sigma Chemical Co.

## Authors' contributions

RMB was responsible for the conception and design of the study, interpretation of the data, and drafting the manuscript. DRW participated in the conception and design of the study and carried out the binding studies. JMS participated in data analysis and manuscript preparation. BJS and DMS participated in the conception and interpretation of the study. CFM was responsible for experimental design, statistical analyses, data interpretation and drafting the manuscript. All authors read and approved the final manuscript.

## Figures and Tables

**Table 1:** Competition analysis (cold saturation) of [^3^H]RX821002 sites in hippocampal homogenates from male and female pups that were exposed to IV saline or cocaine in utero. (Means ± SEM). n = 14 per group.

		K_d _(nM)	B_MAX _(fmol/mg protein)	n_H_
CONTROL	Male	0.8 ± 0.1	84.8 ± 14.1	0.94 ± 0.05
	Female	1.3 ± 0.6	115.4 ± 10.7	0.97 ± 0.09
COCAINE	Male	0.8 ± 0.1	134.1 ± 16.7	0.98 ± 0.11
	Female	1.2 ± 0.2	125.3 ± 10.7	1.09 ± 0.16
